# Foam Roller Post-High-Intensity Training for CrossFit Athletes: Does It Really Help with Recovery?

**DOI:** 10.3390/jfmk10010091

**Published:** 2025-03-11

**Authors:** Fernando Zarzosa-Alonso, Alejandra Alonso-Calvete, Martín Otero-Agra, María Fernández-Méndez, Felipe Fernández-Méndez, Francisco Martín-Rodríguez, Roberto Barcala-Furelos, Myriam Santos-Folgar

**Affiliations:** 1 Faculty of Education and Sports Sciences, Univerity of Vigo, 36005 Pontevedra, Spain; fzarzosa@uvigo.es; 2 Faculty of Physiotherapy, University of Vigo, 36005 Pontevedra, Spain; alejalonso@uvigo.es; 3 REMOSS Research Group, University of Vigo, 36005 Pontevedra, Spain; martinoteroagra@gmail.com (M.O.-A.); mariajosefernandezmendez@gmail.com (M.F.-M.); fernandez.mendez.felipe@gmail.com (F.F.-M.); m.santos.folgar@gmail.com (M.S.-F.); 4 School of Nursing of Pontevedra, University of Vigo, 36005 Pontevedra, Spain; 5 Center for Advanced Clinical Simulation, Advanced Life Support Unit, Emergency Medical Services, Faculty of Medicine, University of Valladolid, 47005 Valladolid, Spain; fmartin@saludcastillayleon.es; 6 Department of Obstetrics, Complexo Hospitalario of Pontevedra, Sergas, 36002 Pontevedra, Spain

**Keywords:** lactate, fatigue, CrossFit, heart rate, rating of perceived effort, workout of the day

## Abstract

**Background/Objectives**: Foam rolling is a new and emergent recovery tool in sports. The aim of this study seeks to compare the acute effects of foam rolling and passive rest on recovery markers in CrossFit athletes following a high-intensity workout. **Methods**: A quasi-experimental crossover study design was completed with 14 amateur CrossFit athletes. Participants completed the FRAN CrossFit WOD and then completed a recovery protocol including either a foam roller or passive rest. Heart rate, blood lactate, and perceived exertion were collected at three time points (pre-workout, post-WOD, and post-recovery). **Results**: The foam rolling group had no significant differences from the passive rest group on recovery markers. Blood lactate increased significantly post-WOD in both groups, yet there was no significant difference in blood lactate post-recovery. Perceived exertion and heart rate had a similar pattern to blood lactate. **Conclusions**: High-intensity training causes significant physiological changes; however, foam rolling offers no additional benefit over passive rest for lactate clearance, heart rate recovery, and perceived exertion. CrossFit athletes can choose their preferred method of recovery based on personal preference, as foam rolling neither enhances nor hinders recovery.

## 1. Introduction

CrossFit^®^ (CrossFit, Inc., Washington, DC, USA) is a high-intensity functional fitness modality [1] that has gained significant popularity due to its diverse range of exercises performed at high intensity. Characterized by constant variation, functional movements, and high intensity through metabolic conditioning, gymnastics, and weightlifting [2], CrossFit challenges individuals to develop a broad fitness capacity. The demanding nature of CrossFit training elicits various physiological adaptations, including increased metabolic rate, improved cardiovascular capacity, and enhanced muscular development. The workouts require athletes to perform tasks with little to no rest, aiming to complete them as quickly as possible (for time) or achieve the highest number of repetitions in a specific time frame (AMRAP). This high-intensity approach enhances muscular endurance, strength, overall fitness, and body composition [3,4,5,6].

The metabolic impact of high-intensity CrossFit exercise can take between 48 and 72 h to return to a baseline state [7], and includes significant changes in blood lactate concentrations right after training, as analyzed in prior and recent research [8,9,10]. Therefore, the assessment of recovery in athletes who perform high-intensity training is of great importance, in order to optimize training monitoring and minimize potential harmful effects associated with performing repeated high-intensity CrossFit^®^ sessions [7].

Adequate recovery is essential to optimize performance and prevent injuries in this type of training. Several recovery methods have been described in CrossFit, and for this reason, various recovery strategies have been studied in the field of high-intensity training, such as low-intensity leg pedaling or NMES to the lower limbs [11]. However, to our knowledge, there are no studies that have analyzed the effect of foam rolling as a self-myofascial release technique, in which the goal is musculature rolled and compressed [12], has emerged as a popular recovery tool among CrossFit athletes.

Foam rollers have been suggested to improve various performance parameters and to accelerate recovery. Thus, the use of a foam roller has become a popular intervention in various sports environments, both to improve training efficiency and competition preparation, as well as to accelerate post-exercise recovery [13]. These improvements have been detailed in lifeguards [14] or soccer players [15], but the effects on recovery appear to be controversial, with significant but small effects on delayed onset muscle soreness and blood lactate clearance [13].

The foam roller is a common element in CrossFit boxes, and its use is widespread. However, to our knowledge, no evidence has been published on the effects of foam rolling on the recovery of CrossFit athletes. To address this knowledge gap, this study aims to compare the acute effects of foam rolling and passive rest on recovery markers, such as blood lactate and perceived exertion, in CrossFit athletes after a high-intensity workout.

## 2. Materials and Methods

**Figure 1:**
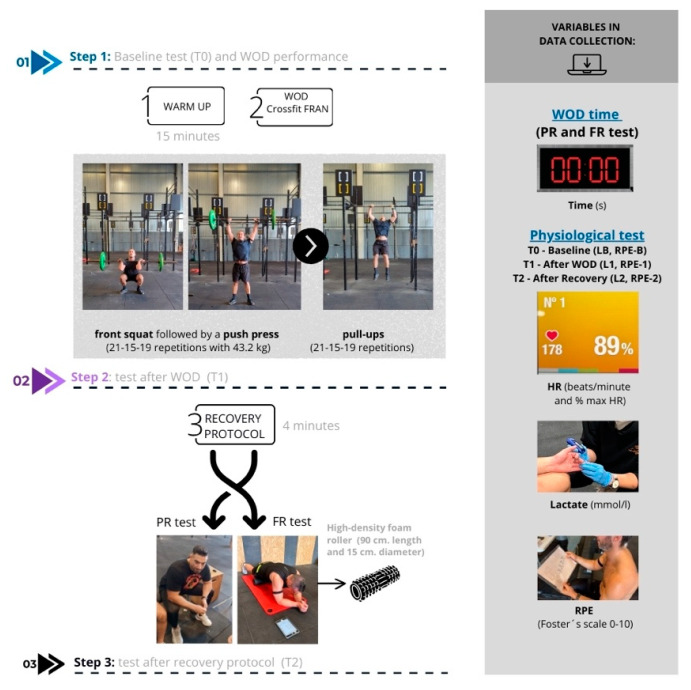
Flow chart design. Workout of the day (WOD); passive recovery test (PR); foam rolling test (FR); baseline test (T0); after WOD test (T1); after recovery test (T2); heart rate (HR); rating of perceived effort (RPE).

A quasi-experimental crossover design was developed in order to analyze lactate concentrations, blood glucose levels, heart rate, and RPE in fourteen amateur CrossFit athletes. The entire procedure of this study can be found in the flow chart in Figure 1.

### 2.1. Sample

Fourteen athletes (13 men and 1 woman) were recruited for this study. Their age and anthropometric characteristics were an age of 36 years [29–44 IQR], a weight of 79 kg [72–87 IQR], a height of 176 cm [172–181 IQR], and 4.5 years performing CrossFit [4–9 IQR]. The inclusion criteria required all participants to be CrossFit athletes with more than two years of experience and at least 3 days of training per week. Subjects did not use any recovery method or follow any specific dietary recommendation. All participants conducted a CrossFit WOD and then recovered with either a foam roller intervention or resting. This investigation was approved by the Ethics Committee of the Faculty of Nursing of Pontevedra (University of Vigo) with code (01-1024) and performed according to the Declaration of Helsinki. Before this study, subjects were informed about all the interventions and procedures and signed a written informed consent form.

### 2.2. Intervention and Procedures

This study was performed by athletes from Box 004 fitness center, located in the city of Pontevedra (Spain). The environmental conditions during the intervention were carefully monitored. The temperature ranged between 15 °C and 17 °C, with a relative humidity varying from 89% to 93%. Data collection took place on two consecutive Saturdays, November 9th and November 16th, during 2024.

All athletes recruited for this study performed the same intervention based on a standard dynamic warm-up protocol with mobility and specific strength exercises. Then, the FRAN CrossFit WOD was selected based on previous studies [16,17]. This CrossFit benchmark WOD was a combination of barbell thrusters (a front squat followed by a push press with 43.2 kg), and pull-ups performed in a 21-15-9 repetition scheme, where athletes performed 21 thrusters and 21 pull-ups, then 15 thrusters and 15 pull-ups, then 9 thrusters and 9 pull-ups as fast as possible [18]. Variations of pull-ups, including butterfly and kipping, were valid.

The measurements were taken prior to the workout (pre-warm-up) and then 3 min after finishing the WOD and just after finishing the recovery protocol (foam roller or resting), according to previous studies with these devices [10,11,14,15].

### 2.3. Recovery

In this research, 2 recovery modalities were tested to study their influence on blood lactate, perceived exhaustion, and heart rate (mean and maximum). Foam rolling (FR) has been suggested as an appropriate recovery tool after the FRAN WOD, and passive recovery (PR) was chosen as a control situation. All subjects were instructed in foam rolling before the test. After the WOD, participants began the recovery protocol. The 2 situations were performed by each participant in randomized order, with a total intervention time of 4 min.

#### 2.3.1. Passive Recovery

During PR, participants remained rested and seated during the whole duration of the recovery period (4 min).

#### 2.3.2. Foam Roller Recovery

The FR consisted of rolling with the device on quadriceps of both legs. Participants were instructed on this method prior to the intervention. They had to stay first in a prone position, locate the device on the upper area of the quadriceps, and then compress the muscle while moving across the muscle belly. The muscle was rolled in two sets: 60 s of rolling each leg and rolling again. The final time of the recovery protocol was 4 min. In order to standardize the amount of pression exerted, a VAS scale was used and a pressure level from 6 to 7 was required of the participants during the intervention [19,20].

For this intervention, a high-density foam roller was used (90 cm length and 15 cm diameter).

### 2.4. Variables

The following variables were recorded both during the PR and FR: (1) time, (2) heart rate (HR), (3) blood lactate, and (4) rating of perceived effort (RPE).

1. Time (in seconds) was measured using Polar App software version 3.5.8 (Polar Verity Sense and Polar Team App; Polar Electro OY) on an iPad (iOS Stopwatch) and recorded at three time points: after warm-up (T0), after WOD (T1), and after the recovery protocol (T2).
2. HR was monitored using a heart rate monitor (Polar Verity Sense and Polar Team App; Polar Electro OY) and recorded as beats per minute (bpm) and as a percentage of maximum heart rate.
3. Blood lactate measurements were taken at three different moments: under basal conditions (LB), 3 min after the WOD (L1), and after the recovery protocol (L2). Lactate measurements were assessed with a capillary device (LactateScout, SensLab GmbH, Leipzig, Germany) and expressed in mmol/L, with an accuracy of ±3% (minimum standard deviation: ±0.2 mmol/L). The first drop of blood was discarded in all measurements.
4. The RPE was measured using Foster’s scale [21] before the warm-up (RPE basal), after the WOD (RPE 1), and after recovery (RPE 2) in four areas: global, legs, arms, and cardio.

### 2.5. Statistical Analysis

All analyses were conducted using IBM SPSS Statistics version 21 for Windows (Armonk, NY, USA). Variables were described through measures of central tendency (mean or median), measures of dispersion (standard deviation or interquartile range), and confidence estimators (95% confidence intervals) based on the normality of the distributions (assessed using the Shapiro–Wilk test). For the comparison of variables, Student’s *t*-test for related samples or the Wilcoxon signed-rank test for related samples was used (depending on the normality of the distributions) for the comparison of single-measure variables. In the comparison of variables with multiple measures in each of the recovery strategies, repeated measures ANOVA with Bonferroni correction or the Friedman test for repeated measures with Bonferroni correction was employed (depending on the normality of the distributions). In cases where the comparisons were statistically significant (significance level ≤ 0.05), effect size was calculated using Cohen’s d or Rosenthal’s r (depending on the normality of the distributions), using the following classification: trivial < 0.2; small (0.2–0.5); moderate (0.5–0.8); large (0.8–1.3); very large (>1.3).

## 3. Results

### 3.1. WOD Duration

The average time taken to complete the WOD was 317 s [interquartile range (IQR) 242–370] in the PR group and 288 s [IQR 242–422] in the FR group. Although participants in the FR group tended to complete the WOD slightly faster, no significant differences were found between these times (*p* = 0.33).

### 3.2. Physiological Variables

#### 3.2.1. Heart Rate

HR results are detailed in Table 1. The mean HR during the recovery protocols was 152 ± 16 bpm (CI95%: 142–161) for PR and 158 ± 11 bpm (CI95%: 152–165) for FR. No significant differences were found between these groups (*p* = 0.09). The maximum HR registered in this moment was 172 ± 12 bpm (CI95: 166–179) for PR and 176 ± 7 bpm (CI95%: 172–180) for FR, with no significant differences between them (*p* = 0.06). However, significant differences were found in HR at the end of the recovery protocols (*p* = 0.002; ES = 1.22, large).

In the intra-group analysis, a significant decrease in HR was found from T0 (before the WOD) to T2 (after recovery) both in PR (*p* < 0.001; ES = 2.23, very large) and in FR (*p* < 0.001; ES = 3,27, very large).

#### 3.2.2. Blood Lactate

Results regarding blood lactate are shown in Table 2. In the PR group, blood lactate levels in T0 were 1.6 mmol/L (IQR 1.2–2.1), in T1 were 13.9 mmol/L (IQR 13.3–18.1), and in T2 were 14.9 mmol/L (IQR 13.6–16.8). Significant differences were reported between T0 and T1 (*p* < 0.001; ES = 0.76, moderate) and between T0 and T2 (*p* < 0.001; ES = 0.76, moderate). No significant differences were observed between T1 and T2 (*p* > 0.05).

In the FR group, blood lactate levels in T0 were 1.8 mmol/L (IQR 1.3–2.3), in T1 were 16.7 mmol/L (IQR 15.0–17.9), and in T2 were 15.3 mmol/L (IQR 13.0–18.3). Significant differences were found between T0 and T1 (*p* < 0.001; ES = 0.92, large) and between T0 and T2 (*p* = 0.002; ES = 0.73, moderate). No significant differences were observed between T1 and T2 (*p* > 0.05).

In an intra-group comparison, no significant differences were observed between any time moments (*p* > 0.05).

#### 3.2.3. Perceived Exertion (RPE)

Results of the RPE are detailed in Table 3.

In the intra-group comparison, no significant differences were found between FR and PR in any measurement of the RPE scale (*p* > 005).

In general RPE, significant differences were found between T0 and T1 (*p* < 0.001; ES = 0.86, large) and between T1 and T2 (*p* = 0.015; ES = 0.62, moderate) in the PR group, and only between T0 and T1 (*p* < 0.001; ES = 0.95, large) in the FR group.

In leg RPE, significant differences were found between T0 and T1 in both the PR (*p* = 0.005; ES = 0.68, moderate) and FR group (*p* < 0.001; ES = 0.85, large).

In arm RPE, significant differences were found between T0 and T1 (*p* < 0.001; ES = 0.85, large) in the PR group, and between T0 and T1 (*p* < 0.001; ES = 0.91, large) and T1 and T2 (*p* = 0.031; ES = 0.58, moderate) in the FR group.

In cardio RPE, significant differences were found between T0 and T1 (*p* < 0.001; ES = 1.05, large) and between T1 and T2 (*p* = 0.015; ES = 0.62, moderate) in the PR group, and only between T0 and T1 (*p* < 0.001; ES = 1.02, large) in the FR group.

## 4. Discussion

This study aimed to examine the effect of foam rolling on recovery from a high-intensity CrossFit workout by comparing it to passive recovery. The main findings were as follows: (a) high-intensity training (Fran’s WOD) produces significant changes in HR, blood lactate levels, and perceived fatigue and (b) the use of a foam roller did not result in any improvements or deficits in physiological recovery of lactate or perceived fatigue.

Regarding HR, this variable significantly decreased post-recovery in both the (PR) and (FR) groups, but there was no significant difference between the groups. This suggests that foam rolling does not provide any additional benefit in modulating cardiac autonomic recovery compared to simple rest. Foam rolling primarily acts on the local musculature, targeting myofascial release [22]. Its influence on systemic cardiovascular parameters like HR is likely indirect and minimal [23,24]. PR, on the other hand, allows for autonomic downregulation and the gradual return of parasympathetic dominance [25], which may be just as effective for HR recovery.

Regarding blood lactate, both PR and FR groups experienced a substantial rise in lactate levels post-WOD due to the anaerobic demands of the FRAN workout [2]. However, lactate levels did not significantly decrease after recovery, regardless of the method. Lactate clearance relies on systemic mechanisms such as increased oxygen delivery, enzymatic activity, and hepatic conversion of lactate to glucose [20,26,27]. According to this explanations and in agreement with previous research, foam rolling may not be sufficient to stimulate these processes to a significant extent [19,27]. Moreover, the 4 min foam rolling protocol might have been too short to facilitate meaningful lactate removal. An important consideration is the timing of sample collection, as it may have influenced lactate measurements. Lactate dynamics fluctuate rapidly post-exercise, with peak concentrations occurring shortly after cessation of activity, followed by a gradual clearance phase [20,26,27]. If sample collection did not capture these fluctuations adequately, it may have affected the interpretation of recovery efficiency. Active recovery protocols, such as low-intensity cycling, have shown superior lactate clearance, as they involve sustained aerobic activity [13,26].

About the perceived exertion measured with RPE, no significant difference was observed between FR and PR in reducing perceived exertion across the measured domains (global, legs, arms, and cardio). This finding agrees with the previous variables analyzed (HR and blood lactate), with no differences between the type of recovery. The RPE scale is a subjective metric influenced by psychological and physiological factors [28,29]. While foam rolling is often perceived as relaxing, the short duration or specific muscle focus (quadriceps) may have limited its impact on overall fatigue perception. Furthermore, individual preferences, tolerance for foam rolling pressure, or psychological factors (e.g., placebo effect) might also influence RPE outcomes.

In recent years, foam rolling has been integrated into the training routines of athletes, particularly in high-intensity sports like CrossFit, with the aim of improving performance and expediting recovery [13]. This accessibility, affordability, and time efficiency of foam rolling devices [30] have contributed to its widespread adoption, especially in high-intensity exercise. For this reason, this device has garnered great interest in CrossFit, where training with rapid increases in HR and stimulation of the anaerobic metabolism result in significant lactate accumulation [2]. The duration and intensity of such exercise directly influence the physiological response, determining the magnitude and rate of lactate accumulation, and the rapid clearance of lactate post-exercise is crucial for recovery and subsequent performance [2]. Nevertheless, the results of this study suggest that FR has no extra effects in comparison with a PR. Several reasons have been previously described, including the possible underlying physiological effects, but the possible placebo effect should be considered, since FR provides an active recovery and movement is necessary, exerting pressure over the device, but PR offers complete rest, which may influence the perception of athletes.

In summary, high-intensity exercise protocols, commonly employed in modalities like CrossFit, elicit significant physiological responses, including elevated heart rate and lactate accumulation. The perception of fatigue is closely linked to these metabolic changes. While foam rolling has been proposed as a recovery strategy to mitigate these effects, further research is needed to fully elucidate the mechanisms underlying its benefits. Future studies should investigate the optimal timing, duration, and pressure of foam rolling interventions to maximize their impact on post-exercise recovery and performance. Moreover, research on other promising methods such as blood flow restriction could be of great interest to these athletes.

### 4.1. Practical Implications

The practical implications of this study impact CrossFit practitioners and their coaches, providing valuable information for both the structure of post-WOD recovery and investment in equipment for training centers. In a digital world where much of the information in the fitness field is obtained through non-scientific channels (Instagram influencers, YouTubers, TikTokers) that offer biased information or are sponsored by brands or laboratories, scientific research becomes increasingly necessary, even if the findings are not favorable.

### 4.2. Study Limitations

This study has several limitations that should be highlighted. First, the sample is small and local; a different type of sample, a different athlete profile, and broader diversity in terms of sex, age, or training level could yield different results. This study measured acute recovery, but the medium-term effects (24/48 h) of the different types of recovery are still unknown. More studies are needed to expand the scientific evidence on the use of foam rollers in CrossFit.

## 5. Conclusions

High-intensity training causes significant physiological variations in HR, blood lactate levels, and perceived fatigue. However, cardiac recovery, lactate clearance, or perceived fatigue based on foam rolling immediately post-training does not provide any benefit or harm compared to passive recovery. Therefore, each athlete can choose to incorporate this element or not based on their personal preference.

## Figures and Tables

**Table 1:** Heart Rate variables (N = 14).

	Passive Recovery (PR)			Foam Rolling Recovery (FR)			*p*-Value (ES)
Variables	T0 (Before WOD)	T1 (WOD)	T2 (After Recovery)	T0 (Before WOD)	T1 (WOD)	T2 (After Recovery)	
Heart rate (bpm)	78 ± 17 (68–88)	-	108 ± 9 (104–113)	79 ± 15 (70–88)	-	119 ± 10 (114–125)	T0 vs. T2 PR: *p* < 0.001 (2.38) FR: *p* < 0.001 (3.27) PR vs. FR T0: *p* = 0.86 T2: *p* = 0.002 (1.22)
Max heart rate (bpm)	-	172 ± 12 (166–179)	-	-	176 ± 7 (172–180)	-	PR vs. FR *p* = 0.06
Mean heart rate (bpm)	-	152 ± 16 (142–161)	-	-	158 ± 11 (152–165)	-	PR vs. FR *p* = 0.09

Results described in mean ± standard deviation and 95% confidence intervals in pairs. Comparisons by repeated measures ANOVA test with Bonferroni correction (*p* ≤ 0.05). Effect size with Cohen’s d test: trivial < 0.2; small (0.2–0.5); moderate (0.5–0.8); large (0.8–1.3); very large (>1.3).

**Table 2:** Lactate variable (N = 14).

Variable	Passive Recovery (PR)			Foam Rolling Recovery (FR)			*p*-Value (ES)
	T0 (Before WOD)	T1 (WOD)	T2 (After Recovery)	T0 (Before WOD)	T1 (WOD)	T2 (After Recovery)	
Lactate (mmol/L)	1.6 (1.2–2.1)	13.9 (13.3–18.1)	14.9 (13.6–16.8)	1.8 (1.3–2.3)	16.7 (15.0–17.9)	15.3 (13.0–18.3)	Passive recovery T0 vs. T1: *p* = 0.001 (0.76) T0 vs. T2: *p* = 0.001 (0.78) T1 vs. T2: *p* = 1.00 Foam Rolling recovery T0 vs. T1: *p* < 0.001 (0.92) T0 vs. T2: *p* = 0.002 (0.73) T1 vs. T2: *p* = 1.00 Passive recovery vs. Foam Rolling T0: *p* = 1.00 T1: *p* = 1.00 T2: *p* = 1.00

Results described in median and interquartile range in pairs. Comparisons by repeated measures Friedman test with Bonferroni correction (*p* ≤ 0.05). Effect size with Rosenthal’s d test: trivial < 0.2; small (0.2–0.5); moderate (0.5–0.8); large (0.8–1.3); very large (>1.3).

**Table 3:** RPE variables (N = 14).

Variable	Passive Recovery (PR)			Foam Rolling Recovery (FR)			*p*-Value (ES)
	T0 (Before WOD)	T1 (WOD)	T2 (After Recovery)	T0 (Before WOD)	T1 (WOD)	T2 (After Recovery)	
General RPE	4 (3–7)	9 (8–10)	6 (5–8)	3 (1–5)	8 (8–9)	7 (5–7)	Passive recovery T0 vs. T1: *p* < 0.001 (0.86) T0 vs. T2: *p* = 1.00 T1 vs. T2: *p* = 0.015 (0.62) Foam Rolling recovery T0 vs. T1: *p* < 0.001 (0.95) T0 vs. T2: *p* = 0.30 T1 vs. T2: *p* = 0.10 Passive vs. Foam Rolling T0: *p* = 1.00 T1: *p* = 1.00 T2: *p* = 1.00
Legs RPE	4 (2–6)	8 (7–8)	6 (4–7)	3 (2–3)	8 (7–9)	5 (5–7)	Passive recovery T0 vs. T1: *p* = 0.005 (0.68) T0 vs. T2: *p* = 1.00 T1 vs. T2: *p* = 0.13 Foam Rolling recovery T0 vs. T1: *p* < 0.001 (0.85) T0 vs. T2: *p* = 1.00 T1 vs. T2: *p* = 0.051 Passive vs. Foam Rolling T0: *p* = 1.00 T1: *p* = 1.00 T2: *p* = 1.00
Arms RPE	2 (2–3)	9 (8–10)	6 (3–7)	2 (1–3)	8 (6–9)	7 (4–7)	Passive recovery T0 vs. T1: *p* < 0.001 (0.85) T0 vs. T2: *p* = 1.00 T1 vs. T2: *p* = 0.051 Foam Rolling recovery T0 vs. T1: *p* < 0.001 (0.91) T0 vs. T2: *p* = 0.031 (0.58) T1 vs. T2: *p* = 1.00 Passive vs. Foam Rolling T0: *p* = 1.00 T1: *p* = 1.00 T2: *p* = 1.00
Cardio RPE	0 (0–2)	9 (7–10)	5 (3–5)	0 (0–2)	9 (8–9)	6 (3–7)	Passive recovery T0 vs. T1: *p* < 0.001 (1.05) T0 vs. T2: *p* = 0.35 T1 vs. T2: *p* = 0.015 (0.62) Foam Rolling recovery T0 vs. T1: *p* < 0.001 (1.02) T0 vs. T2: *p* = 0.07 T1 vs. T2: *p* = 0.15 Passive vs. Foam Rolling T0: *p* = 1.00 T1: *p* = 1.00 T2: *p* = 1.00

Results described in median and interquartile range in pairs. Comparisons by Repeated measures Friedman test with Bonferroni correction (*p* ≤ 0.05). Effect size with Rosenthal’s d test: trivial < 0.2; small (0.2–0.5); moderate (0.5–0.8); large (0.8–1.3); very large (>1.3).

## Data Availability

The original contributions presented in this study are included in the article. Further inquiries can be directed to the corresponding author(s).
